# Association of triglyceride-glucose index and high-sensitivity C-reactive protein with contrast-induced nephropathy after percutaneous coronary intervention in patients with acute coronary syndrome: a retrospective cohort study

**DOI:** 10.3389/fcvm.2026.1733377

**Published:** 2026-02-11

**Authors:** Guowei Tian, Caiyuan Qian, Zhihao Liu, Ye Xu, Li Yang, Liqun He

**Affiliations:** 1 Department of Cardiology, Intervention Cardiology Center, Wuhan No.1 Hospital, Wuhan, China; 2 Department of Nephrology, Renmin Hospital of Wuhan University, Wuhan, China

**Keywords:** acute coronary syndrome, contrast-induced nephropathy, high-sensitivity C-reactive protein, interaction effect, percutaneous coronary intervention, triglyceride-glucose index

## Abstract

**Background:**

Contrast-induced nephropathy (CIN) remains a frequent and serious complication after percutaneous coronary intervention (PCI) in acute coronary syndrome (ACS) patients. The triglyceride-glucose (TyG) index, a marker of insulin resistance, and high-sensitivity C-reactive protein (hs-CRP), an inflammatory biomarker, may contribute to CIN development.

**Methods:**

This retrospective cohort study included 1,818 ACS patients undergoing PCI between 2017 and 2024. Logistic regression, restricted cubic splines (RCS), and interaction analyses were performed to examine the nonlinear relationships and additive effects of TyG index and hs-CRP on CIN risk.

**Results:**

Multivariate logistic regression analysis indicated that both the TyG index (OR = 4.08, 95% CI: 2.95–5.71) and hs-CRP levels (OR = 1.07, 95% CI: 1.05–1.10) were significant independent risk factors for CIN (all *P* < 0.001). The RCS analysis revealed nonlinear associations between the TyG index, hs-CRP, and CIN risk (*P* < 0.001). Threshold effect analysis identified optimal cutoff values of TyG index ≥8.5 and hs-CRP ≥5.0 mg/L, beyond which CIN risk sharply increased. Patients with both TyG index ≥8.5 and hs-CRP ≥5.0 mg/L showed the highest CIN risk (adjusted OR = 8.356, 95% CI: 2.13–32.8), with significant additive interactions observed (RERI = 4.914, A*P* = 0.588, SI = 3.012). The predictive model demonstrated a robust area under the ROC curve (AUC = 0.780, 95% CI: 0.750–0.809), with sensitivity of 77.7% and specificity of 69.3%.

**Conclusion:**

Elevated TyG index and hs-CRP levels independently and synergistically increase CIN risk in ACS patients after PCI. These findings highlight the interplay between metabolic and inflammatory pathways in CIN pathogenesis and may help identify high-risk individuals for early preventive strategies.

## Introduction

Contrast-induced nephropathy (CIN) is one of the most frequent complications following percutaneous coronary intervention (PCI) (1, 2). CIN not only prolongs hospital stays but can also lead to increased medical expenses, an elevated risk of renal replacement therapy, recurrent vascular reconstruction procedures, and increased long-term mortality (3, 4). Therefore, identifying risk factors for CIN and implementing effective preventive measures are of paramount importance.

Insulin resistance (IR) constitutes a crucial pathogenic mechanism in diabetes mellitus (DM) and significantly influences both cardiovascular disease incidence and patient prognosis, while also being closely associated with the prevalence of chronic kidney dysfunction (5–7). The triglyceride-glucose (TyG) index is a novel and easily accessible biomarker for IR (8). Prior studies have suggested a potential association between an elevated TyG index and CIN development in specific populations, such as patients with ST-elevation myocardial infarction (STEMI) (5, 9, 10).

Furthermore, inflammation is recognized as one of the primary pathophysiological mechanisms underlying CIN (11). High-sensitivity C-reactive protein (hs-CRP) serves as an important biomarker of systemic inflammatory response (5). Previous studies have established an association between elevated hs-CRP levels and post-PCI CIN, identifying it as an independent risk factor (1, 12). However, current research has primarily focused on STEMI patient populations, with limited evidence regarding whether these conclusions can be extended to acute coronary syndrome (ACS) patients, and the presence of interactive effects remains unclear (4, 5).

This study aims to investigate the individual and combined associations of the TyG index and hs-CRP with CIN in ACS patients undergoing PCI. By analyzing their independent associations, potential interactive effects, and exploring optimal thresholds for risk stratification, we seek to provide an integrated biomarker approach to help identify high-risk patients and guide targeted preventive strategies.

## Materials and methods

### Study design and population

**Figure 1:**
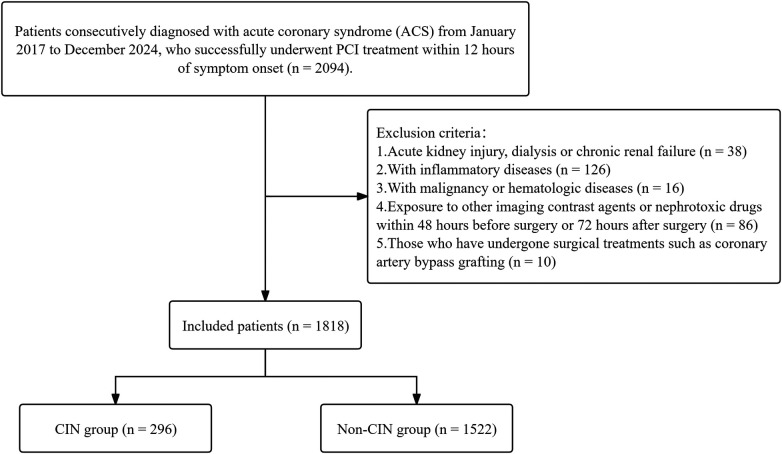
Patient selection flowchart. CIN, contrast-induced nephropathy.

This retrospective cohort study included consecutive patients diagnosed with ACS, encompassing ST-segment Elevation Myocardial Infarction (STEMI), Non-ST-segment Elevation Myocardial Infarction (NSTEMI), and unstable angina (UA), between January 2017 and December 2024. Inclusion criteria were: successful primary PCI [Thrombolysis in Myocardial Infarction (TIMI) flow grade ≥2] within 12 h of symptom onset with complete clinical data. Exclusion criteria included: (1) pre-admission acute kidney injury, dialysis or chronic kidney failure [estimated glomerular filtration rate (eGFR) < 30 mL·min^−^¹·1.73 m^−^²], totaling 38 patients; (2) inflammatory diseases (such as active pulmonary infection, intestinal inflammation, or autoimmune diseases), totaling 126 patients; (3) malignant tumors or hematologic diseases, 16 patients; (4) exposure to other imaging contrast agents or nephrotoxic drugs within 48 h pre-procedure or 72 h post-procedure, 86 patients; (5) previous cardiac bypass surgery, 10 patients. Ultimately, a total of 1,818 consecutive patients meeting study criteria were included. Figure 1 illustrates the participant selection process.

### Ethics statement

This study adhered to the ethical requirements of the Declaration of Helsinki and received approval from the Ethics Committee of xxx Hospital (approval number:). Due to the retrospective nature of this study using anonymized data, informed consent requirements were waived. All procedures were conducted according to the Declaration of Helsinki and relevant regulations, with data security and participant privacy protected through strict confidentiality agreements and data encryption measures.

### Definitions and measurements

Blood samples were collected via antecubital vein. All laboratory tests were performed on blood samples collected prior to coronary angiography (CAG). Fasting plasma glucose (FPG), triglycerides (TG), SCr, and other biochemical parameters were measured using a Beckman Coulter AU 5,800 automated analyzer (Beckman Coulter Inc., CA, USA). SCr was measured within 48 h post-procedure to monitor for CIN development. The TyG index was calculated using the following formula: TyG = ln[fasting TG [mg/dL] × FPG [mg/dL]/2] (13).

CIN was defined as an increase in SCr >25% or 0.5 mg/dL from baseline within 48 to 72 h after contrast agent use (14). The eGFR was calculated using the Modification of Diet in Renal Disease equation (15, 16). Body mass index (BMI) was calculated as weight (kg) divided by height squared (m²). DM diagnostic criteria included: (1) history of DM or current use of antidiabetic medications; (2) FPG ≥126 mg/dL; (3) random plasma glucose ≥200 mg/dL; (4) 2-hour oral glucose tolerance test value ≥200 mg/dL; (5) glycated hemoglobin (HbA1c) ≥ 6.5% (17). Hypertension diagnostic criteria were: (1) history of hypertension diagnosis or current use of antihypertensive medications; (2) systolic blood pressure (SBP) ≥ 140 mmHg and/or diastolic blood pressure (DBP) ≥ 90 mmHg (18). Blood pressure was measured using a mercury sphygmomanometer three times after 5 min of patient rest, with the average value taken. Ejection fraction (EF) was determined by two experienced cardiologists using the Simpson method with an EPIQ 7 echocardiography device (Philips Medical Systems, Chicago, USA).

### Coronary angiography and PCI procedure

CAG was performed by experienced cardiologists using Siemens CAG equipment (Siemens Healthineers, GmbH, Erlangen, Germany). Eligible patients received PCI according to European Society of Cardiology (ESC) guidelines (19). All CAG procedures used iohexol-containing contrast agents. The number of diseased vessels (stenosis ≥50%), contrast volume used (units: cc), number of patients receiving PCI, number of stents implanted, and total stent length were recorded. All patients received loading doses of aspirin (300 mg) and clopidogrel (600 mg) or ticagrelor (180 mg) before surgery (20). Additionally, during PCI, all patients received unfractionated heparin at a dose of 100 U/kg and were managed according to a standardized prophylactic hydration protocol (intravenous infusion of 0.9% sodium chloride at a rate of 0.5–1 mL/kg/h for 4 h before and after the procedure; a rate of 0.5 mL·kg^−^¹·h^−^¹ was recommended for patients with NYHA class III/IV). However, in patients undergoing primary PCI for ST-segment elevation myocardial infarction or requiring urgent intervention because of hemodynamic instability, preprocedural hydration could be omitted or shortened to prioritize reperfusion therapy. In such cases, hydration therapy was initiated and completed as early as possible after the procedure (21, 22).

### Statistical analysis

All data analyses were performed using R 4.3.2 statistical software. Normally distributed data are presented as mean ± standard deviation (SD), non-normally distributed data as median (p25, p75), and categorical data as percentages (%). Data normality was assessed using the Kolmogorov–Smirnov test. For normally distributed data, one-way analysis of variance (ANOVA) was used for group comparisons; for non-normally distributed data, the Kruskal–Wallis H test was employed. Categorical data group differences were analyzed using the chi-square test.

Univariate logistic regression analysis was initially employed to screen variables with *p*-values < 0.1 for entry into the candidate pool. Subsequently, a multivariable logistic regression model was constructed using a dual-strategy approach: (1) variables with univariate *p* < 0.1 were included as candidates, and (2) clinically established predictors—specifically diabetes mellitus, LVEF, eGFR, and age—were forced into the final model based on prior clinical knowledge, existing literature consensus, and their inclusion in validated risk-scoring systems such as the Mehran contrast-induced nephropathy risk score, irrespective of their univariate p-values. The final model was refined using backward stepwise elimination with a removal threshold of *p* ≥ 0.05; however, the clinically mandated variables were retained throughout the selection process to ensure clinical interpretability and alignment with established pathophysiological frameworks. Due to high collinearity between the TyG index, FPG, and TG, FPG and TG were not included in the multivariate logistic regression analysis. Receiver operating characteristic (ROC) curves were used to evaluate the predictive performance of the multivariate logistic regression model. To assess potential overfitting and estimate the model's generalizability, we performed internal validation using the bootstrap resampling method with 2,000 replicates. The optimism-corrected area under the ROC curve (AUC) and its 95% confidence interval were calculated. Optimism, defined as the difference between the apparent performance (on the original dataset) and the average performance on bootstrap test sets, was reported to quantify overfitting.

To further explore the association between CRP and the TyG index with CIN, restricted cubic spline (RCS) models were employed with adjustment for multivariate significant variables. Threshold effects were determined through segmental effect analysis and node calculation. The additive interaction between the TyG index and hs-CRP in CIN occurrence was evaluated, specifically analyzing the interrelationships between different TyG index and hs-CRP level groups. Adjusted odds ratios (aOR) and their 95% confidence intervals (CI) were used to demonstrate the effects of the TyG index and hs-CRP on CIN occurrence. Additive interaction was assessed through relative excess risk due to interaction (RERI), attributable proportion (AP), and synergy index (SI). When RERI > 0, AP > 0, or SI > 1, positive additive interaction was indicated (23).

Statistical significance was set at *p* < 0.05.

## Results

A total of 1,818 patients were included in this study, of whom 296 developed CIN, while 1,522 did not develop CIN. Baseline data showed that patients in the CIN group had significantly higher BMI, heart rate, peak hs-CRP, total cholesterol, TG, low-density lipoprotein cholesterol (LDL-C), FPG, and calcium channel blocker (CCB) treatment usage rates, while eGFR levels were significantly lower (*p* < 0.05). Furthermore, analysis by ACS subtype revealed that the incidence of CIN was significantly higher in STEMI patients (58%) than in those with NSTEMI (26%) or unstable angina (UA) (17%) (*p* < 0.05). Detailed data are shown in Table 1.

**Table 1:** Baseline characteristics of patients with contrast-induced nephropathy (CIN) and Non-contrast-induced nephropathy (Non-CIN).

Variable	CIN (*n* = 296)	Non-CIN (*n* = 1,522)	*P value*
Age (years)	62.07 (54.13, 69.29)	62.72 (53.94, 72.15)	0.32
Sex			0.20
Male	232 (78%)	1,137 (75%)	
Female	64 (22%)	385 (25%)	
BMI (kg/m²)	26.34 (25.34, 27.27)	24.90 (22.81, 26.79)	<0.001*
Hypertension	145 (49%)	761 (50%)	0.80
Diabetes mellitus	38 (13%)	171 (11%)	0.49
Smoking history	166 (56%)	839 (55%)	0.81
Family history of CAD	27 (9%)	164 (11%)	0.46
Previous ACS	8 (3%)	41 (3%)	>0.99
Chronic kidney disease	6 (2%)	49 (3%)	0.36
LVEF (%)	54.74 (49.92, 58.39)	54.20 (49.97, 58.49)	0.41
Killip class ≥2	45 (15%)	293 (19%)	0.12
GRACE score	137.03 (121.03, 146.48)	136.30 (127.60, 146.98)	0.30
Heart rate (bpm)	84.08 (81.25, 86.45)	82.99 (79.66, 85.93)	<0.001*
Systolic BP (mmHg)	122.42 (119.61, 126.74)	123.50 (119.71, 129.41)	0.44
Diastolic BP (mmHg)	79.84 (78.01, 81.53)	79.97 (77.50, 82.25)	0.66
WBC count (×10⁹/L)	9.24 (6.93, 11.87)	9.65 (7.33, 12.30)	0.09
RBC count (×10¹²/L)	4.52 (4.43, 4.62)	4.51 (4.41, 4.64)	0.14
Hemoglobin (g/L)	139.41 (136.95, 141.90)	139.58 (136.46, 143.10)	0.93
Neutrophil count (×10⁹/L)	7.86 (5.59, 10.62)	7.50 (5.14, 10.25)	0.02*
Lymphocyte count (×10⁹/L)	1.48 (1.14, 1.97)	1.48 (1.15, 1.98)	0.57
Monocyte count (×10⁹/L)	0.47 (0.36, 0.59)	0.44 (0.34, 0.61)	0.13
Platelet count (×10⁹/L)	219.97 (185.26, 263.76)	222.91 (184.81, 259.01)	0.79
Peak troponin T (ng/L)	485.45 (244.54, 994.92)	529.13 (244.69, 941.20)	0.67
Peak CK-MB (U/L)	97.78 (44.53, 139.71)	98.59 (46.88, 135.08)	0.52
Peak NT-proBNP (pg/mL)	1,124.83 (593.58, 2,149.00)	1,183.50 (580.47, 2,187.49)	0.79
Peak hs-CRP (mg/L)	6.40 (5.05, 8.20)	4.50 (3.30, 5.60)	<0.001*
Total cholesterol (mmol/L)	4.61 (4.06, 5.13)	4.39 (3.85, 4.87)	<0.001*
Triglycerides (mmol/L)	1.66 (1.36, 1.96)	1.47 (1.10, 1.85)	<0.001*
HDL cholesterol (mmol/L)	0.98 (0.86, 1.08)	1.02 (0.89, 1.13)	0.08
LDL cholesterol (mmol/L)	3.02 (2.65, 3.42)	2.85 (2.39, 3.30)	<0.001*
TyG index	9.23 (9.02, 9.46)	8.94 (8.61, 9.24)	<0.001*
eGFR (mL/min/1.73m²)	91.10 (77.34, 106.10)	93.89 (78.62, 108.91)	0.035
Blood urea nitrogen (mmol/L)	5.98 (5.73, 6.22)	5.99 (5.66, 6.35)	0.23
Fasting blood glucose (mg/dL)	140.17 (124.18, 159.24)	121.75 (104.81, 138.12)	<0.001*
Uric acid (µmol/L)	337.69 (313.30, 354.57)	340.67 (320.32, 356.40)	0.46
Multivessel disease	84 (28%)	438 (29%)	0.95
LCX lesion	170 (57%)	841 (55%)	0.53
LAD lesion	41 (14%)	195 (13%)	0.69
RCA lesion	136 (46%)	682 (45%)	0.77
Procedure duration (minutes)	51.85 (49.36, 57.03)	52.81 (49.74, 57.92)	0.46
Contrast volume (mL)	145.85 (140.80, 148.55)	146.67 (143.72, 148.91)	0.07
No reflow phenomenon	45 (15%)	232 (15%)	>0.9
IABP use	6 (2%)	25 (2%)	0.82
Aspirin therapy	287 (97%)	1,481 (97%)	0.89
P2Y12 inhibitor	292 (99%)	1,479 (97%)	0.21
Statin therapy	274 (93%)	1,447 (95%)	0.11
Beta blocker therapy	244 (82%)	1,265 (83%)	0.84
CCB therapy	66 (22%)	250 (16%)	0.02*
ACEI/ARB therapy	148 (50%)	754 (50%)	0.94
Nitrate therapy	45 (15%)	296 (19%)	0.10
Heparin therapy	190 (64%)	955 (63%)	0.69
Diuretic therapy	183 (62%)	871 (57%)	0.16
ACS subtypes			<0.001*
STEMI	171 (58%)	720 (47%)	
NSTEMI	76 (26%)	342 (22%)	
UA	49 (17%)	460 (30%)	

CIN, contrast-induced nephropathy; Non-CIN, non-contrast-induced nephropathy; BMI, body mass index; ACS, acute coronary syndrome; CAD, coronary artery disease; LVEF, left ventricular ejection fraction; GRACE, global registry of acute coronary events; BP, blood pressure; WBC, white blood cell; RBC, red blood cell; hs-CRP, high-sensitivity C-reactive protein; NT-proBNP, N-terminal pro B-type natriuretic peptide; TyG index, triglyceride-glucose index; eGFR, estimated glomerular filtration rate; IABP, intra-aortic balloon pump; CCB, calcium channel blocker; ACEI/ARB, angiotensin-converting enzyme inhibitor/angiotensin II receptor blocker; ACS, acute coronary syndrome; STEMI, ST-segment elevation myocardial infarction; NSTEMI, Non-ST-segment elevation myocardial infarction; UA, unstable angina.

* Statistically significant.

Univariate and multivariate regression analyses showed that BMI (1.17, 95% CI 1.12–1.23, *p* < 0.001), heart rate (1.02, 95% CI 1.00–1.04, *p* = 0.011), peak hs-CRP (1.07, 95% CI 1.05–1.10, *p* < 0.001), total cholesterol (1.46, 95% CI 1.22–1.75, *p* < 0.001), LDL-C (1.61, 95% CI 1.31–1.99, *p* < 0.001), and the TyG index (4.08, 95% CI 2.95–5.71, *p* < 0.001) were all independent risk factors (see Table 2).

**Table 2:** Univariate and multivariate logistic regression analysis for contrast-induced nephropathy.

Variable	Univariable OR (95% CI, *p*-value)	Multivariable OR (95% CI, *p*-value)
Age	1.00 (0.99–1.01, *p* = .360)	0.99 (0.98–1.01, *p* = .073)
Sex	0.81 (0.60–1.10, *p* = .180)	
BMI	1.15 (1.11–1.20, *p* < .001)	1.17 (1.12–1.23, *p* < .001)
Hypertension	0.96 (0.75–1.23, *p* = .750)	
Diabetes mellitus	1.16 (0.80–1.69, *p* = .429)	1.16 (0.76–1.73, *p* = .479)
Smoking history	1.04 (0.81–1.34, *p* = .762)	
Family history CAD	0.83 (0.54–1.27, *p* = .397)	
Previous ACS	1.00 (0.47–2.16, *p* = .993)	
Chronic kidney disease	0.62 (0.26–1.47, *p* = .278)	
LVEF	0.99 (0.97–1.01, *p* = .377)	0.98 (0.96–1.00, *p* = .073)
Killip class ≥2	0.75 (0.53–1.06, *p* = .102)	
GRACE score	1.00 (0.99–1.00, *p* = .285)	
Heart rate	1.02 (1.01–1.03, *p* = .002)	1.02 (1.00–1.04, *p* = .011)
Systolic BP	1.00 (0.99–1.01, *p* = .504)	
Diastolic BP	1.00 (0.99–1.02, *p* = .707)	
WBC count	0.97 (0.94–1.01, *p* = .096)	0.97 (0.93–1.01, *p* = .099)
RBC count	0.82 (0.60–1.14, *p* = .239)	
Hemoglobin	1.00 (0.99–1.01, *p* = .943)	
Neutrophil count	1.04 (1.01–1.07, *p* = .008)	1.02 (0.99–1.05, *p* = .229)
Lymphocyte count	0.95 (0.80–1.13, *p* = .594)	
Monocyte count	0.70 (0.40–1.19, *p* = .188)	
Platelet count	1.00 (1.00–1.00, *p* = .776)	
Peak troponin T	1.00 (1.00–1.00, *p* = .672)	
Peak CK-MB	1.00 (1.00–1.00, *p* = .535)	
Peak NT-proBNP	1.00 (1.00–1.00, *p* = .801)	
Peak hs-CRP	1.07 (1.05–1.09, *p* < .001)	1.07 (1.05–1.10, *p* < .001)
Total cholesterol	1.39 (1.18–1.63, *p* < .001)	1.46 (1.22–1.75, *p* < .001)
HDL cholesterol	0.61 (0.32–1.16, *p* = .134)	
LDL cholesterol	1.58 (1.32–1.91, *p* < .001)	1.61 (1.31–1.99, *p* < .001)
TyG index	3.75 (2.79–5.04, *p* < .001)	4.08 (2.95–5.71, *p* < .001)
eGFR	0.99 (0.99–1.00, *p* = .050)	0.99 (0.99–1.00, *p* = .080)
Blood urea nitrogen	0.95 (0.86–1.05, *p* = .290)	
Uric acid	1.00 (1.00–1.00, *p* = .472)	
Multivessel disease	0.98 (0.74–1.29, *p* = .889)	
LCX lesion	1.09 (0.85–1.41, *p* = .491)	
LAD lesion	1.09 (0.76–1.57, *p* = .626)	
RCA lesion	1.05 (0.82–1.34, *p* = .719)	
Procedure duration	1.00 (0.99–1.01, *p* = .493)	
Contrast volume	0.98 (0.97–1.00, *p* = .072)	0.98 (0.97–1.00, *p* = .087)
No reflow phenomenon	1.00 (0.70–1.41, *p* = .986)	
IABP USE	1.24 (0.50–3.05, *p* = .641)	
Aspirin therapy	0.88 (0.42–1.84, *p* = .739)	
P2Y12 inhibitor	2.12 (0.76–5.96, *p* = .153)	
Statin therapy	0.65 (0.39–1.06, *p* = .082)	0.65 (0.39–1.13, *p* = .125)
Beta-blocker therapy	0.95 (0.69–1.32, *p* = .775)	
CCB therapy	1.46 (1.08–1.98, *p* = .015)	1.17 (0.83–1.63, *p* = .364)
ACEI/ARB therapy	1.02 (0.79–1.31, *p* = .885)	
Nitrate therapy	0.74 (0.53–1.05, *p* = .088)	0.98 (0.66–1.44, *p* = .928)
Heparin therapy	1.06 (0.82–1.38, *p* = .638)	
Diuretic therapy	1.21 (0.94–1.56, *p* = .143)	

BMI, body mass index; ACS, acute coronary syndrome; CAD, coronary artery disease; LVEF, Left ventricular ejection fraction; GRACE, global registry of acute coronary events; BP, blood pressure; WBC, white blood cell; RBC, red blood cell; hs-CRP, high-sensitivity C-reactive protein; NT-proBNP, N-terminal Pro B-type natriuretic peptide; TyG index, Triglyceride-glucose index; eGFR, estimated glomerular filtration rate; IABP, intra-aortic balloon pump; CCB, calcium channel blocker; ACEI/ARB, angiotensin-converting enzyme inhibitor/angiotensin II receptor blocker.

**Figure 2:**
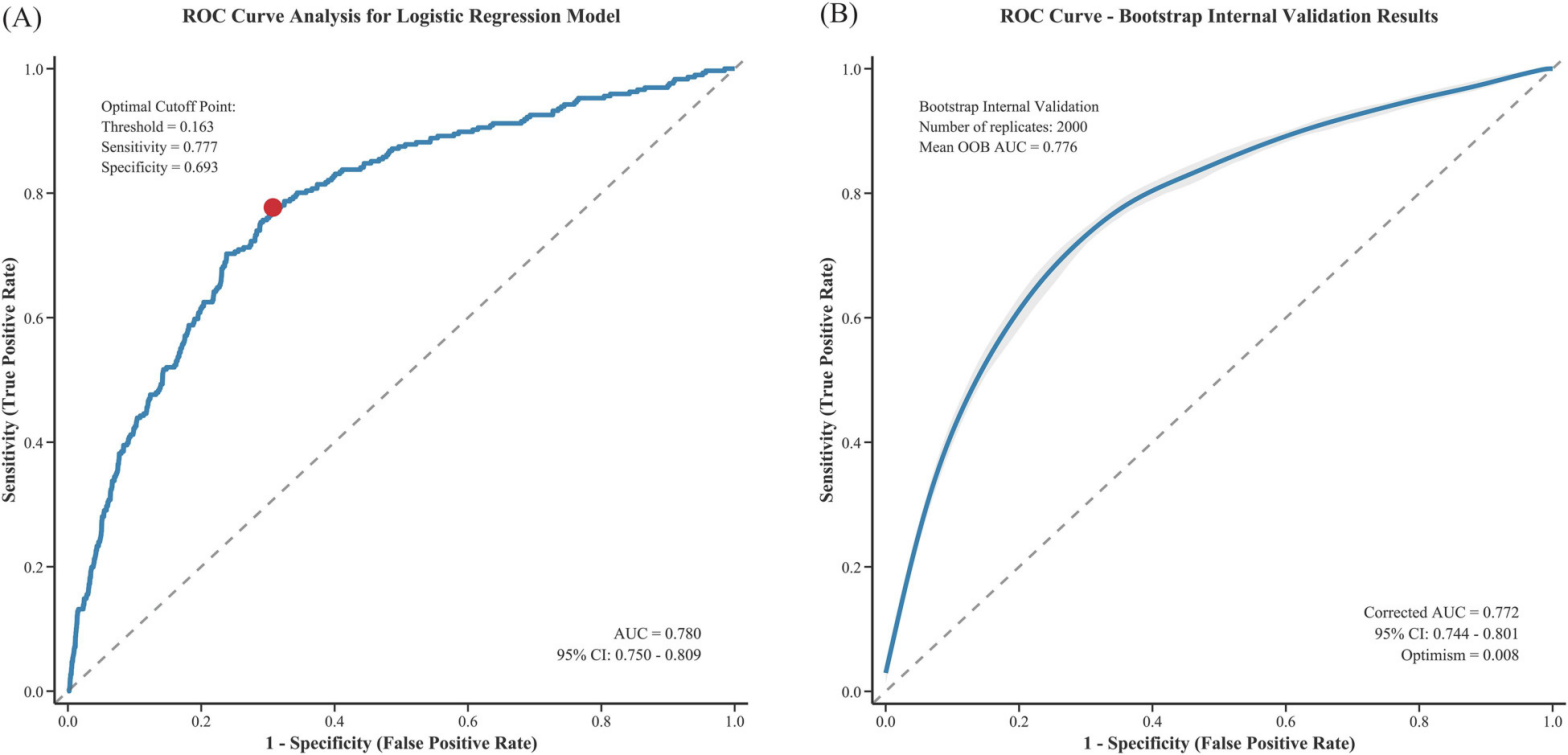
ROC curve analysis of the logistic regression model for predicting CIN after PCI: **(A)** original ROC curve and **(B)** bootstrap internal validation.

The multivariable logistic regression model incorporating both statistically significant predictors and clinically mandated variables demonstrated good discriminatory ability, with an area under the ROC curve (AUC) of 0.780 (95% CI 0.750–0.809). At the optimal cutoff point of 0.163, the model achieved a sensitivity of 77.7% and a specificity of 69.3% (Figure 2A). After bootstrap internal validation, the optimism-corrected AUC was 0.772 (95% CI 0.744–0.801) with an optimism of 0.008, indicating minimal overfitting (Figure 2B). Additionally, variance inflation factor (VIF) collinearity testing showed all variables had VIF values <4, indicating no collinearity issues (Supplementary Table 1).

**Figure 3:**
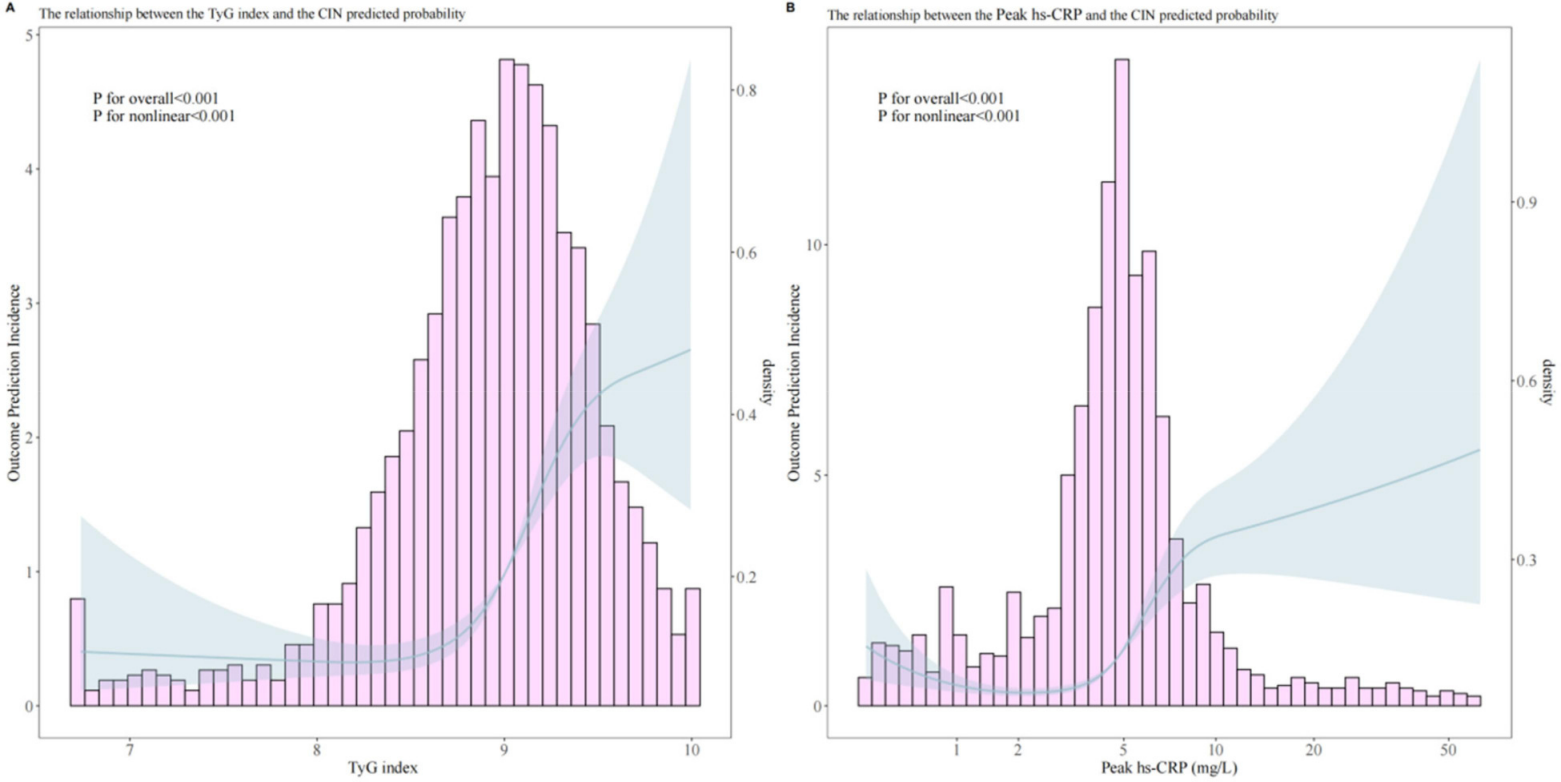
Analysis of the relationship between predicted probability of post-PCI CIN and TyG index **(A)**, peak hs-CRP **(B)**.

RCS analysis revealed significant nonlinear relationships between the TyG index and peak hs-CRP with CIN occurrence (*P* for nonlinear < 0.001). Threshold effect analysis of the TyG index and peak hs-CRP showed significant correlations for both (Figure 3).

Overall analysis results indicated a significant association between the TyG index and CIN occurrence (OR = 3.861, 95% CI 2.825–5.346, *p* < 0.001). Through threshold analysis (Table 3), the optimal cutoff point for the TyG index was 8.5. When the TyG index was <8.5, its effect on CIN was weak (OR = 1.171, 95% CI 0.581–2.813, *p* = 0.690); when the TyG index was >8.5, its effect on CIN was significantly enhanced (OR = 5.117, 95% CI 3.527–7.473, *p* < 0.001).

**Table 3:** Threshold effect analysis for TyG Index and peak hs-CRP.

Variable	Model	Analysis	OR (95% CI)	*P* value
TyG index	Overall association		3.861 (2.825–5.346)	< 0.001
	Threshold analysis	Cutpoint: 8.5		
		< 8.5	1.171 (0.581–2.813)	0.690
		> 8.5	5.117 (3.527–7.473)	< 0.001
		LRT *P* value		0.007
Peak hs-CRP	Overall association		3.009 (2.335–3.913)	< 0.001
	Threshold analysis	Cutpoint: 5.0 mg/L		
		< 5.0 mg/L	2.248 (1.417–3.746)	0.001
		> 5.0 mg/L	3.599 (2.490–5.242)	< 0.001
		LRT *P* value		0.187

TyG, triglyceride-glucose index; hs-CRP, high-sensitivity C-reactive protein; OR, odds ratio; CI, confidence interval; LRT, likelihood ratio test.

Peak hs-CRP overall analysis also showed significant association (OR = 3.009, 95% CI 2.335–3.913, *p* < 0.001), with an optimal cutoff point of 5.0 mg/L. When peak hs-CRP was <5.0 mg/L, it was associated with increased CIN risk (OR = 2.248, 95% CI 1.417–3.746, *p* = 0.001); when peak hs-CRP was >5.0 mg/L, the risk was further increased (OR = 3.599, 95% CI 2.490–5.242, *p* < 0.001).

### Interaction analysis

Interaction analysis of the TyG index and peak hs-CRP showed that when both were at high levels (TyG index ≥8.5 and hs-CRP ≥5 mg/L), the risk of CIN occurrence was significantly increased (aOR = 8.356, 95% CI 2.13–32.8). The RERI for this interaction was 4.914, AP was 0.588, and SI was 3.012, all indicating strong additive interaction. Additionally, the low-high combination risk of the TyG index and peak hs-CRP was lower than the double-high group. Comprehensive analysis indicates significant additive interaction between the TyG index and hs-CRP in CIN pathogenesis, jointly increasing risk.

Table 4 Assessment of Additive Interaction Between TyG Index and hs-CRP on Incident CIN.

**Table 4:** Assessment of the additive interaction between TyG index and hsCRP on incident AKI.

Groups	aOR (95% CI)	Additive interaction measures		
	aOR (95% CI)	RERI (95% CI)	AP (95% CI)	SI (95% CI)
Low TyG index (< 8.5) & Low hsCRP (< 5 mg/L)	1.0 (ref)	–	–	–
Low TyG index (< 8.5) & High hsCRP (≥ 5 mg/L)	2.363 (1.04–5.37)	–	–	–
High TyG index (≥ 8.5) & Low hsCRP (< 5 mg/L)	2.079 (1.01–4.28)	–	–	–
High TyG index (≥ 8.5) & High hsCRP (≥ 5 mg/L)	8.356 (2.13–32.8)	4.914 (2.768–12.005)	0.588 (0.331–0.919)	3.012 (1.595–12.160)

aOR, adjusted odds ratio; RERI, relative excess risk due to interaction; AP, attributable proportion; SI, synergy index. Models were adjusted for age, sex, BMI, hypertension, diabetes, and other relevant covariates. RERI > 0, AP > 0, or SI > 1 indicates positive additive interaction.

## Discussion

This study identified multiple independent risk factors for CIN development following PCI through univariate and multivariate regression analyses, including BMI, heart rate, peak hs-CRP, total cholesterol, LDL-C, and the TyG index. These results indicate that CIN occurrence stems from multiple factors involving metabolic, inflammatory, and hemodynamic aspects. This study identified relevant thresholds for the TyG index and hs-CRP in predicting CIN following PCI in ACS patients using restricted cubic spline (RCS) and threshold effect analyses, thereby contributing to the refinement of clinical risk stratification. Additionally, a significant additive interaction exists between the TyG index and hs-CRP, suggesting synergistic effects of metabolic and inflammatory factors in CIN development. These findings provide important evidence for understanding CIN pathogenesis and developing prevention strategies.

Previous studies have extensively demonstrated that IR adversely affects kidney function and prognosis, also increasing CIN risk following elective PCI (6, 24, 25). The TyG index, as a novel IR biomarker in recent years, offers advantages of easy accessibility, low cost, and simple operation. Previous research has already revealed the negative impact of an elevated TyG index on kidney function (26). Aktas H demonstrated that in non-diabetic non-STEMI patients, a high TyG index similarly increases post-procedural CIN incidence (8). Similarly, Gursoy E found that a high TyG index was associated with increased CIN risk in patients with similar inclusion criteria (20). This indicates that the TyG index is a risk factor for CIN (27). Consistent with previous studies, our research confirms the importance of the TyG index and hs-CRP as CIN risk factors. Similarly, hs-CRP as a systemic inflammation indicator has been found to be a predictor of post-PCI CIN, especially in patient populations with high inflammatory levels (11), which is consistent with this study. Threshold analysis in this study identified two cut-off values with clear clinical applicability: a TyG index ≥ 8.5 and hs-CRP ≥ 5 mg/L. Patients meeting both criteria exhibited a further increased risk of CIN compared with those with elevation of either marker alone, suggesting a synergistic effect of metabolic dysfunction and inflammatory status in the development of CIN. These findings underscore the need for heightened clinical vigilance in this patient subgroup.

The mechanisms by which the TyG index and hs-CRP contribute to the development of CIN after PCI in patients with ACS have not yet been fully elucidated. As a representative surrogate marker of IR, the TyG index has been clearly shown to increase the risk of cardiovascular disease and DM (28, 29). Evidence indicates that a hyperglycemic milieu promotes oxidative stress through multiple pathways, including the formation of advanced glycation end products, activation of protein kinase C, mitochondrial dysfunction, and stimulation of the polyol pathway. These processes enhance the generation of reactive oxygen species, leading to endothelial dysfunction, vascular inflammation, and aggravated cardiovascular injury. Moreover, oxidative stress disrupts nitric oxide signaling, impairs vasodilation, and promotes vasoconstriction. Oxidative stress and inflammation can directly induce microcirculatory dysfunction caused by atherosclerotic plaque formation and thrombosis (30, 31). In addition, IR exerts deleterious effects on renal function by increasing glomerular hydrostatic pressure, enhancing glomerular permeability, and ultimately resulting in glomerular hyperfiltration (32–34). The role of hs-CRP in the development of CIN may involve multiple mechanisms. Previous studies have demonstrated a close association between glucose metabolism and inflammation (35–37). Reports suggest that this process may be related to the production of renal inflammatory cytokines and chemokines, upregulation of leukocyte adhesion molecules, and infiltration of various inflammatory cells into renal tissue (38). The interaction between these two mechanisms is likely attributable to the mutual reinforcement between disordered glucose metabolism (such as IR) and chronic low-grade inflammation (as reflected by elevated hs-CRP levels), forming a vicious cycle. IR adversely affects renal function, whereas inflammatory mediators—such as tumor necrosis factor-α (TNF-α) and interleukin-6 (IL-6)—further impair insulin signaling pathways, thereby exacerbating IR. This metabolic–inflammatory synergy may jointly damage the renal microcirculation, increase oxidative stress, and reduce renal tolerance to contrast media toxicity, ultimately contributing to an increased risk of CIN (39, 40).

Notably, in the baseline analysis of this study, the median contrast volume was slightly lower in patients who developed CIN than in those who did not (*P* = 0.07), which appears inconsistent with the established role of contrast volume as a classic risk factor. We believe this finding most likely reflects risk-adaptive management in real-world practice. Operators make procedural decisions based on patients’ real-time clinical information, among which baseline renal function (eGFR) is a key determinant. In our cohort, baseline eGFR was significantly lower in the CIN group (*P* = 0.035). Consequently, operators may have consciously adopted stricter contrast minimization strategies in patients identified as having poorer baseline renal function and higher risk. This risk-based, differential treatment may have introduced a “confounding by treatment” effect, leading to an apparently lower contrast volume in the high-risk group. This interpretation is supported by the multivariable analysis, in which contrast volume did not demonstrate independent predictive value after adjustment for confounders, including BMI and total cholesterol.

The strengths of this study are twofold. First, this cohort study, based on a relatively large sample size, identified a synergistic effect of the TyG index and hs-CRP on the risk of CIN in patients with ACS undergoing PCI, with the robustness of the findings supported by internal validation. Second, clinically relevant risk thresholds for both indicators were identified, which may facilitate improved clinical risk stratification. Several limitations should also be acknowledged. First, this was a single-center study predominantly involving an Asian population, and patients with impaired renal function were excluded, which may limit the generalizability of the findings. Second, due to the retrospective design, causal inferences cannot be established and require confirmation in prospective studies. Third, the TyG index and hs-CRP were measured only once before PCI, precluding evaluation of their dynamic changes over time. Fourth, despite multivariable adjustment, residual confounding from unmeasured factors such as lifestyle and dietary habits cannot be excluded. Fifth, inter-operator variability in coronary angiography and PCI procedures may have influenced outcomes. Sixth, interaction analyses resulted in small sample sizes and limited event numbers in some subgroups, potentially leading to unstable estimates. Finally, in emergency settings of acute myocardial infarction, standardized fasting blood sampling was not feasible in some patients, which may have affected laboratory parameters sensitive to feeding status.

In conclusion, this study confirmed that BMI, heart rate, hs-CRP, cholesterol parameters, and the TyG index are independent risk factors for post-PCI CIN. The nonlinear and additive interactions between the TyG index and hs-CRP highlight the importance of metabolic-inflammatory interactions. These findings provide theoretical support for optimizing CIN risk prediction and developing personalized prevention strategies, opening new directions for future research and clinical practice.

## Data Availability

Data are available upon request to the corresponding author. Requests to access these datasets should be directed to Liqun He, dyyyxxgnk123@163.com.
