# Supplementary material for: Association of triglyceride-glucose index and high-sensitivity C-reactive protein with contrast-induced nephropathy after percutaneous coronary intervention in patients with acute coronary syndrome: a retrospective cohort study
Source: Front Cardiovasc Med. 2026 Feb 11;13:1733377. doi: 10.3389/fcvm.2026.1733377 (PMC12932434; doi:10.3389/fcvm.2026.1733377)
Supplement: Supplementary file 1 [file Table1.docx]

**Supplement Material**

| **Supplementary Table 1. Variance Inflation Factor (VIF) Analysis for Multicollinearity Assessment** | | |
| --- | --- | --- |
| **Variable** | **VIF Value** | **Interpretation** |
| BMI | 1.021 | Low multicollinearity |
| Heart_rate | 1.055 | Low multicollinearity |
| Peak_hs_CRP | 1.048 | Low multicollinearity |
| Diabetes_mellitus | 1.003 | Low multicollinearity |
| LVEF | 1.030 | Low multicollinearity |
| Age | 1.025 | Low multicollinearity |
| eGFR | 1.024 | Low multicollinearity |
| Total_cholesterol | 1.018 | Low multicollinearity |
| LDL_cholesterol | 1.031 | Low multicollinearity |
| TyG_index | 1.035 | Low multicollinearity |
